# Effectiveness of a Teaching Program on Ebola Virus Knowledge Among Nursing Students

**DOI:** 10.7759/cureus.82832

**Published:** 2025-04-23

**Authors:** Vishal K Ghorpade, Bahubali J Geddugol

**Affiliations:** 1 Medical Surgical Nursing, Bharati Vidyapeeth (Deemed to be University) College of Nursing, Sangli, IND; 2 Psychiatric Nursing, Bharati Vidyapeeth (Deemed to be University) College of Nursing, Sangli, IND

**Keywords:** assess, ebola virus, effect, nursing students, planned teaching programme

## Abstract

Introduction

Ebolaviruses are negative-stranded RNA viruses in the *Filoviridae* family. They are transmitted to humans through direct contact with the bodily fluids of infected individuals or animals, and they cause severe and often fatal hemorrhagic fever, with mortality rates ranging from 25% to 90%, depending on the outbreak and available healthcare resources. These viruses cause severe systemic disease with high mortality rates. Given the infectious nature of Ebola and the critical role of healthcare providers in managing outbreaks, educating nursing students on the virus's transmission, prevention, and treatment is essential. Enhancing the knowledge of future healthcare professionals can improve healthcare system preparedness, ensuring effective response during outbreaks and reducing the virus's impact.

Methodology

A quasi-experimental one-group pre-test post-test design was utilized to assess the effectiveness of a planned teaching program on Ebola virus knowledge among nursing students. A simple random sampling technique was employed to select participants from nursing colleges in Navi Mumbai. The teaching program, which covered Ebola virus transmission, prevention, and treatment, was administered to the selected students. A pre-test was conducted to assess baseline knowledge, followed by the teaching intervention, and a post-test was performed to measure knowledge gains. Data were analyzed using frequency and percentage distribution and paired t-tests to compare pre-test and post-test scores.

Results

A study was conducted with 50 second-year Bachelor of Science in Nursing students from MGM College of Nursing, Navi Mumbai, to assess the impact of an educational intervention. Participants included 41 females (82%) and nine males (18%), with most living in nuclear families (38 (76%)) and urban areas (45 (90%)). Before the intervention, the pre-test showed that participants' knowledge levels varied: 10 (20%) had average knowledge, eight (16%) had poor knowledge, and 32 (64%) had good knowledge. After the intervention, there was a marked improvement: 32 participants (64%) exhibited excellent knowledge, 16 (32%) had good knowledge, and only two (4%) remained at the average level. This shift highlights the effectiveness of the intervention in enhancing participants’ understanding. Statistical analysis using paired t-tests confirmed a significant increase in knowledge, with no notable associations between demographic variables and pre-test scores.

Conclusion

The study concluded that the planned teaching program significantly enhanced the knowledge of nursing students regarding Ebola virus infection. A notable improvement was observed in the post-test, with most students demonstrating excellent and good knowledge compared to average and poor knowledge levels in the pre-test. These results suggest that targeted educational interventions are effective in improving healthcare students’ understanding of critical infectious diseases like Ebola. Furthermore, no significant associations were found between demographic variables and pre-test knowledge scores, indicating that the teaching program effectively reached all students. The findings underscore the importance of incorporating such teaching programs into nursing curricula to prepare students for managing future public health challenges.

## Introduction

Ebola virus disease (EVD) is a severe illness with high fatality rates, yet healthcare professionals often lack sufficient knowledge about its transmission, prevention, and treatment. While international efforts emphasize EVD training, formal education in the United States remains limited. This study assessed knowledge and attitudes among 269 healthcare students, including pharmacy, nursing, and physician assistant students, using pre- and post-surveys following an educational intervention. Significant knowledge and perception improvements were observed, particularly among pharmacy students, whose attitudes improved notably. This intervention highlights the potential of targeted EVD education to enhance healthcare workers’ preparedness, contributing to better patient care outcomes [1].

Feldmann and Geisbert, in 2011, provide an in-depth review of Ebola haemorrhagic fever, a severe disease caused by Ebolaviruses of the *Filoviridae* family. First identified in 1976, the virus is believed to be zoonotic, with fruit bats as the likely reservoir. Transmission occurs through direct contact with infected bodily fluids or contaminated materials. The disease is characterized by sudden onset, fever, hemorrhaging, and multi-organ failure, with fatality rates ranging from 25% to 90%. The authors discuss the virus’s pathogenesis, immune evasion, and lack of effective treatments or vaccines, stressing the need for improved preparedness and global response strategies [2].

The World Health Organization states that Ebola viruses are primarily transmitted through direct human-to-human contact with infected bodily fluids, contaminated materials, or the bodies of those who have died from the disease. Such transmission often occurs during caregiving or traditional burial practices, where protective measures are limited. These high-risk interactions contribute significantly to the rapid spread of infection during outbreaks, particularly in regions with limited healthcare infrastructure [3].

Izudi and Bajunirwe conducted a comprehensive meta-analysis evaluating the case fatality rate (CFR) of Ebola virus disease (EVD) using global data spanning from 1976 to 2022. Published in the Journal of Infection and Public Health, the study analyzed data from multiple outbreaks and geographic regions, revealing CFRs ranging from 25% to 90%. The findings underscore the variability in fatality rates, which are influenced by factors such as outbreak location, healthcare capacity, and timeliness of intervention. This study highlights the persistent lethality of EVD and the urgent need for improved surveillance, early diagnosis, and robust healthcare infrastructure in affected areas [4].

Their epidemic potential was dramatically highlighted during the 2013-2016 West African outbreak, which resulted in more than 28,000 reported cases and over 11,000 deaths, marking it as the largest and deadliest Ebola outbreak in history. This outbreak was unprecedented in scale, with more than 28,000 confirmed cases and 511,000 deaths [5].

The 2014/2015 EVD outbreak highlighted the need for rapid educational strategies to prepare healthcare professionals. In response, Emory University developed the Just-in-Time Teaching (JiTT) program to address gaps in EVD knowledge, reduce fear, and boost nursing students’ confidence. The program employed informational sessions, self-directed slide presentations, and online resources, evaluated through surveys at multiple time points. Results showed sustained improvements in knowledge and confidence and reduced exposure concerns. This study demonstrates JiTT as an effective, adaptable approach for nursing education during public health crises, providing a model for responding quickly and comprehensively to emerging infectious disease outbreaks [6].

The study by Nwozichi et al. assessed the effectiveness of a self-instructional module (SIM) in improving knowledge of EVD among Nigerian university students in Bengaluru, India. The pre-experimental design revealed a significant improvement in participants' knowledge after the intervention, with pretest and posttest mean scores rising from 16.03 to 28.22, respectively (t = 21.432, p < 0.001). The results highlight the SIM's potential as an effective educational tool for enhancing public health awareness. The authors concluded that SIM should be adopted by health personnel to improve health communication and EVD preparedness [7].

The recurring threat of EVD highlights the need for effective educational interventions to equip healthcare professionals with essential knowledge and preparedness. Studies demonstrate the effectiveness of innovative teaching strategies, such as JiTT and SIMs, in improving EVD-related knowledge, reducing fear, and enhancing confidence among students in healthcare and allied fields. However, there remains a gap in evaluating the specific impact of structured teaching programs (STPs) on nursing students’ understanding of EVD. This study aims to assess the effectiveness of a targeted teaching program in enhancing EVD knowledge among nursing students, addressing a critical need in nursing education.

## Materials and methods

A quasi-experimental research design was chosen to achieve the objectives of this study, as it allows for the assessment of the effectiveness of an intervention without randomization, making it suitable for real-world settings where ethical or logistical constraints limit the use of randomized controlled trials. The primary aim of the study was to evaluate the impact of planned teaching on improving knowledge of EVD among nursing students. The specific objectives included assessing baseline levels of knowledge of EVD, implementing the planned teaching program, and measuring post-intervention changes. The dependent variable was the nursing students’ knowledge of EVD, which reflects the outcome that the research aimed to measure and assess. This variable was expected to change as a result of the intervention. The independent variable was the planned lecture-assisted teaching program, which served as the intervention introduced by the researcher. This educational program was designed to enhance the participants’ understanding of EVD, including its transmission, symptoms, prevention, and management. By examining changes in knowledge levels before and after the implementation of this teaching intervention, the study aimed to evaluate its effectiveness. The relationship between the independent and dependent variables was central to the research, as any observed improvements in knowledge could be attributed to the impact of the structured teaching strategy.

The study was conducted at MGM College of Nursing in Navi Mumbai, as this setting provided access to the target population of nursing students. A total of 50 nursing students were selected as the sample using a non-probability convenience sampling technique. Inclusion criteria consisted of nursing students who were currently enrolled in the program, able to read and comprehend English, and willing to provide informed consent to participate in the study. These criteria ensured that participants could fully engage with the educational content and respond appropriately to the assessment tools. Exclusion criteria included students who were absent during the period of data collection, those who declined to participate or withdrew consent at any stage, and individuals with prior formal training or certification specifically related to EVD, as this could influence baseline knowledge and affect the study's outcomes. The sample size was determined based on a power analysis to ensure adequate statistical power for detecting a significant effect. Using an estimated effect size of 0.5, an alpha level of 0.05, and a power of 0.80, a minimum sample size of 50 participants was calculated to be sufficient. This calculation was performed using G*Power software (Heinrich-Heine-Universität Düsseldorf, Düsseldorf, Germany) to ensure that the study results were reliable and generalizable. The paired t-test was chosen for statistical analysis, as it is appropriate for comparing pre-test and post-test scores within the same group, ensuring the validity and reliability of the findings.

The intervention lasted for about one week and included both the teaching session and the post-test. It was carried out through a planned lecture-assisted teaching program on EVD. The content was based on trusted sources like the World Health Organization (WHO) and the Centers for Disease Control and Prevention (CDC). The session took place in a classroom and used audiovisual tools such as PowerPoint (Microsoft® Corp., Redmond, WA) presentations, posters, and real-life examples to make the learning more effective. Topics covered included the causes of EVD, how it spreads, its signs and symptoms, ways to prevent it, and the role of nurses during outbreaks. A pre-test was given before the session to check students’ knowledge, and a post-test was conducted afterward to measure what they had learned.

The institutional ethics committee of Bharati Vidyapeeth (Deemed to be University), College of Nursing, Sangli, approved the study (IECBVDUCON, Sangli, 16/2023-2024/10/04/2024). Informed consent was obtained from all participants, who were thoroughly informed about the study's objectives, procedures, and their right to withdraw at any time without penalty.

Data were collected using a structured questionnaire called the EVD knowledge assessment tool, which was created for this study to check how much participants knew about EVD. The questionnaire included multiple-choice questions on topics like causes, how the virus spreads, signs and symptoms, prevention, and the role of healthcare workers. It was given before and after the teaching session to see how much the students' knowledge improved. To make sure the tool was accurate, experts in nursing education and infectious diseases reviewed it for content validity. The reliability of the questionnaire was tested using the test-retest method, and it showed a strong score of 0.82, meaning it was consistent and dependable. Data were summarized using frequency and percentage distribution to provide an overview of the participants' demographic characteristics and their responses to the knowledge assessment questions.

The demographic variables of the study population are presented in Appendix A. The structured knowledge questions related to knowledge of Ebola virus are presented in Appendix B.

To determine the statistical significance of changes in knowledge about EVD between the pre-test and post-test assessments, a paired t-test was conducted. This test compared the pre- and post-test scores of the same group of nursing students to assess the effectiveness of the teaching program. The difference between the posttest and pretest scores for each participant was calculated, and the mean of these differences was computed to find the average change in knowledge. The standard deviation of the differences was also calculated to measure the variability of the changes. Using these values, the t-statistic was calculated, the Wilcoxon signed-rank test was also conducted, and the degrees of freedom were determined as the number of participants minus one. The p-value was then obtained based on the t-statistic and degrees of freedom. In this study, a p-value of less than 0.05 was considered statistically significant, indicating that the teaching program had a meaningful impact on the students' knowledge about EVD.

## Results

The study's demographic analysis reveals that most participants are young adults, with 42% aged 18-19 and 44% aged 19-20. A majority (82%) identify as female, while 76% come from nuclear families. Urban residents dominate the sample (90%), and pet ownership is relatively low (24%). Chi-square tests assessed associations between these demographic variables and a particular outcome. Age (χ² = 3.345, p = 0.764), gender (χ² = 2.744, p = 0.254), family type (χ² = 0.966, p = 0.617), locality (χ² = 0.069, p = 0.966), and pet ownership (χ² = 2.577, p = 0.276) all showed no significant associations, as their p-values exceeded the 0.05 threshold. This indicates that none of these variables significantly influenced the outcome, providing valuable context for data interpretation. Understanding the demographic composition and statistical findings helps clarify participant characteristics and their potential impact on study results. The frequency and percent distribution of socio-demographic variables and their association with knowledge are shown in Table 1.

**Table 1:** Frequency, percentage distribution of socio-demographic variables, and their association with knowledge

Variables	Frequency	Percentage	Chi-square test	p-value	Remark
Age (in years)					
18	4	8%	3.345	0.764	No significant association
19	21	42%			
20	22	44%			
Above 20	3	6%			
Gender					
Male	9	18%	2.744	0.254	No significant association
Female	41	82%			
Type of family					
Nuclear	38	76%	0.966	0.617	No significant association
Joint family	12	24%			
Locality					
Urban	45	90%	0.069	0.966	No significant association
Rural	5	10%			
Pet in the home					
Yes	12	24%	2.577	0.276	No significant association
No	38	76%			

**Figure 1:**
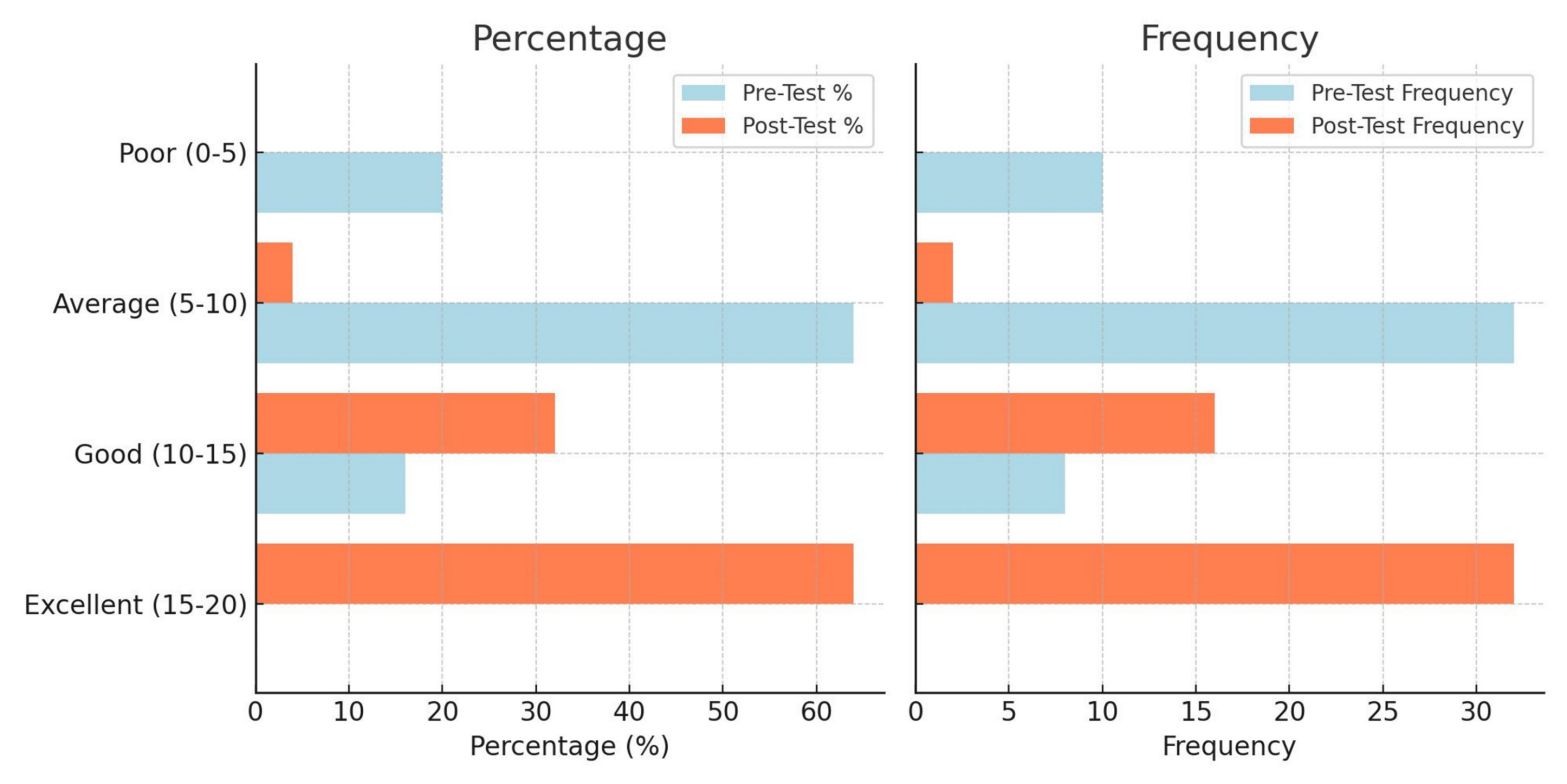
Frequency and percentage distribution of pre-test and post-test knowledge scores

The pre-test results showed that most participants, 32 (64%), had an "average" level of knowledge, while 10 (20%) had "poor" knowledge, and eight (16%) had a "good" understanding. Notably, none of the participants scored in the "excellent" category. However, after the STP, post-test results demonstrated a significant improvement. A majority, 32 (64%), achieved "excellent" scores, while 16 (32%) moved to the "good" category. Only two participants (4%) remained in the "average" range, and none had "poor" scores. A paired t-test (or Wilcoxon signed-rank test) was performed to compare pre-test and post-test scores, revealing a statistically significant improvement (p < 0.05). These findings highlight the effectiveness of the teaching program in enhancing participants' knowledge levels. The frequency and percentage distribution of the pre-test and post-test knowledge scores are shown in Figure 1.

For the pre-test, the average score was 7.3, and the middle value was 7.34, which means most people scored close to 7.3. The spread of scores, shown by the standard deviation of 2.99, tells us that there was some variation in how people performed, with scores being spread out. For the posttest, the average score increased to 15.5, showing a performance improvement. The middle value for the post-test was 17.18, which is higher than the average, suggesting that most people scored higher than the average. The standard deviation of 2.82 is slightly lower than the pre-test, meaning there was less variation in post-test scores. The effect size, as measured by Cohen’s d, was 2.82, indicating a very large impact of the intervention. The 95% confidence interval (1.68-3.96) suggests that the true effect size likely falls within this range, reinforcing the reliability of the findings and confirming the effectiveness of the educational program in significantly improving participants' knowledge levels. The comparison between pre-test and post-test knowledge scores is shown in Table 2.

**Table 2:** Comparison between pre-test and post-test knowledge scores

Test	Mean	Median	Standard deviation	Effect size Cohen’s d	Confidence interval
Pre-test	7.3	7.34	2.99	2.82	95% (1.68-3.96)
Post-test	15.5	17.18	2.82		

## Discussion

The present study aimed to assess nursing students' knowledge regarding Ebola virus infection. The sample primarily consisted of female students aged 19-20 years, living in urban areas, and from nuclear families. The study had three key objectives: to assess pre-test knowledge, determine the effectiveness of an STP, and explore any association between demographic variables and pre-test knowledge scores. The pretest results revealed that students had inadequate knowledge about Ebola, underscoring the need for an effective educational intervention. After implementing the STP, the students' knowledge significantly improved, demonstrating the effectiveness of this teaching method in enhancing their understanding of Ebola and its prevention. Additionally, the study found no significant association between demographic factors such as age, gender, family type, or locality and the pre-test knowledge scores. The lack of a significant association between demographic variables and knowledge scores implies that the intervention may have been the primary driver of knowledge improvement.

The study by Chilton JM et al. explores nursing students' self-reported knowledge of EVD, their willingness to treat EVD patients, and their perceptions of duty to treat. Using a survey tool developed specifically for this purpose, the researchers found that licensed students had significantly higher knowledge of EVD compared to their prelicensure counterparts (p = 0.039). Additionally, students with higher EVD knowledge and older age were more willing to treat patients with EVD. However, their willingness to treat was not influenced by relationship status or whether they had children. The study highlights the importance of EVD education for nursing students to ensure they are prepared to respond effectively during outbreaks. The findings emphasize that beyond basic EVD knowledge, an in-depth understanding of infectious disease principles is essential for ensuring that nurses are ready to treat patients in critical situations [8].

These findings align with previous research by Shivani, emphasizing the role of structured education in improving Ebola-related knowledge among nursing students. Knowledge regarding EVD among nursing students: The effectiveness of an STP on improving knowledge of EVD among second-year Bachelor of Science in Nursing students was assessed. The study, using a pre-experimental research design, found a significant improvement in students' knowledge, with the mean pre-test score of 9.9 rising to 23.9 post-test. The research highlights the importance of educating nursing students on EVD, given its high mortality rate, to ensure they are equipped to care for affected patients and contribute to public health efforts during outbreaks [9].

A study by Eckes et al., educating healthcare providers in the treatment of patients with EVD, discusses the challenges faced by nurses in managing patients with EVD and other virulent infectious diseases. It emphasizes the importance of proper education and training, especially when dealing with diseases that have a limited treatment history and minimal prior patient care experience. The National Institutes of Health Clinical Center developed effective educational strategies to enhance nurses' experiential learning, preparing them to care for patients with EVD. The article highlights the need for increased awareness and precautions when dealing with such dangerous diseases [10].

The study by Otu et al. assessed the effectiveness of a tablet computer tutorial application in improving the knowledge and attitudes of health workers in Ondo State, Nigeria, during the Ebola epidemic. The results demonstrated a significant increase in participants' knowledge of the EVD, including its transmission, symptoms, and prevention methods. The intervention also reinforced positive attitudes toward safety measures such as avoiding contact with infected individuals, safe burial practices, and using personal protective equipment. These findings suggest that mHealth innovations can be a valuable tool in training health workers and strengthening health systems, particularly in resource-limited regions like West Africa, for better epidemic preparedness [11].

The study by Olowookere et al. assessed health workers' preparedness for EVD control and management. It revealed gaps in knowledge and infection control practices among the participants, with only 42% demonstrating good knowledge of EVD. Despite 85.5% being aware of the outbreak, a significant portion lacked awareness about preventive measures, such as the absence of a vaccine. The study highlighted that most workers felt the hospital infection control policies were inadequate, and occupation was the only predictor of good knowledge. The findings underscore the need for improved EVD education and enhanced preparedness in healthcare settings [12].

This study by Ajilore et al. investigates college students' knowledge, attitudes, and adherence to Ebola prevention education delivered through public service announcements (PSAs). The findings reveal a strong awareness of Ebola transmission and prevention, but misconceptions persist. Despite high initial awareness, a significant number of students no longer adhere to the advice in the PSAs, potentially due to the WHO's declaration of Nigeria being Ebola-free. The study highlights the importance of enhancing PSAs to address misconceptions and maintain engagement, offering valuable suggestions for improving future Ebola prevention programs targeted at university students [13].

Taken together, these studies underscore the critical role of structured education and innovative learning strategies in preparing healthcare workers and students for managing Ebola and other infectious diseases. The present study contributes to this growing body of research by reaffirming the effectiveness of targeted educational programs in improving knowledge and preparedness. Future efforts should focus on integrating digital learning tools and continuous training to enhance long-term retention and application of knowledge in clinical practice.

The study was limited to students from a single nursing college in Navi Mumbai, Maharashtra, which means the findings may not be generalizable to nursing students in other regions or countries. The sample size was also relatively small, consisting of only 50 students, which might not fully represent the diverse population of nursing students. Additionally, the study relied on a structured questionnaire to assess knowledge, which may not capture all aspects of students' understanding of the topic. Another limitation is that the study only assessed the immediate impact of the teaching program through a pre-test and post-test without examining long-term retention of knowledge. Future studies could explore the lasting effects of STPs on students' knowledge and behavior. Furthermore, since the study did not include other variables such as the students' prior exposure to Ebola-related information, it is unclear whether their pre-test knowledge levels were influenced by any outside factors. Potential confounders: limitations that prior knowledge might have influenced participants’ responses.

## Conclusions

In conclusion, the present study successfully highlighted the importance of STPs in enhancing the knowledge of nursing students regarding Ebola virus infection. The study findings indicate that before the intervention, the students had inadequate knowledge of Ebola, which is a significant concern considering the crucial role that nursing professionals play in managing and preventing infectious diseases. The results show that after the STP, the students’ knowledge significantly improved, emphasizing the effectiveness of educational interventions in increasing awareness and understanding of critical health issues. This study concludes that implementing STPs is an effective strategy for improving nursing students' understanding of infectious diseases, particularly the Ebola virus. The findings also suggest that regular training and education on emerging health threats should be incorporated into nursing curricula to prepare students to handle such challenges in the future. Overall, the study underscores the need for continued educational efforts to enhance healthcare professionals' knowledge and readiness to manage global health emergencies like Ebola.

## Appendices

Appendix A 

**Table 3:** Demographic variables

Question	Options
1. Age (in years)	a) 17 - 18 b) 18 - 19 c) 19 - 20 d) Above 20
2. Gender	a) Male b) Female
3. Type of Family	a) Nuclear b) Joint
4. Locality	a) Urban b) Rural
5. Is there a pet in your home?	a) Yes b) No
If yes, then which pet?	________
6. Are you aware of the Ebola virus?	a) Yes b) No
If yes, what is the source of information?	a) Magazine b) Newspaper c) Health worker d) Television

Appendix B 

**Table 4:** Structured knowledge questions about Ebola The duly formatted questionnaire used for the assessment is the source of the questionnaire. The questionnaire used for the assessment was developed by Mr. Vishal Ghorpade. It was designed by the author to evaluate nursing students' knowledge of Ebola virus transmission, prevention, and treatment.

Question	Options
1. Who discovered the Ebola virus?	a) Peter Pilot b) Louis Pasteur c) Jonas Salk d) Robert Koch
2. In which country was the Ebola virus first found?	a) African countries b) India c) Russia d) China
3. What is another name for the Ebola virus?	a) Hemorrhagic fever b) Lassa fever c) Yellow fever d) Dengue fever
4. What causes bleeding inside and outside of the body?	a) Measles b) Rabies c) Ebola d) Varicella
5. Which species of fruit bat is responsible for the Ebola virus?	a) Cynopterinae b) Pteropodidae c) Epomophorinae d) Rousettinae
6. The incubation period of the Ebola virus is:	a) 6 to 10 days b) 3 to 4 days c) 2 to 21 days d) 10 to 14 days
7. How does the Ebola virus spread from person to person?	a) By touching b) By blood c) By air-borne transmission d) By insect bites
8. Which of these can pass along the Ebola virus?	a) Dandruff b) Tears c) Fingernails d) Hair
9. What factors contribute to the spread of the Ebola virus?	a) Poverty b) Family history c) Gender d) Climate
10. Choose the symptom that is not associated with Ebola:	a) Bone loss b) Hiccups c) Blindness d) Sore throat
11. Which of the following is a sign of Ebola virus infection?	a) Heart attack b) Hair loss c) Stomach pain d) Weight loss
12. Which are the late symptoms of Ebola virus infection?	a) Prostration b) High temperature c) Loss of appetite d) Nausea
13. How is Ebola virus infection diagnosed?	a) Polymerase chain reaction b) Biopsy c) X-ray d) Sonography
14. What specimens are collected to diagnose the Ebola virus?	a) Blood b) Hair c) Skin biopsy d) Nails
15. Which of the following is NOT a complication of the Ebola virus?	a) Dyspnea b) Shock c) Fatigue d) John’s Syndrome
16. Which drug is used to reduce the mortality rate of Ebola virus infection?	a) Antiviral drugs b) Paracetamol c) Gelusil d) Diclofenac
17. How can you prevent the Ebola virus?	a) By vaccination b) By weight control c) By proper nutrition d) By exercise
18. Which vaccine is used for the Ebola virus?	a) BCG b) MMR c) rVSV-ZEBOV d) ACAM2000
19. What precautions should be taken while handling an infected person?	a) Use PPE b) Exercise regularly c) Sleep well d) Get vaccinated
20. What is the management for systematic complications of the Ebola virus?	a) IV fluids b) Blood sugar monitoring c) Physiotherapy d) Phototherapy
